# Sarcoma of Femur: Amputation

**Published:** 1887-12

**Authors:** 


### SARCOMA OF FEMUR: AMPUTATION.

CASE II.

G—— S——, aged 18 years, was admitted on November 10th, with painful swelling of the left knee. The patient's general health and family history are good. He gave the following history:

Six weeks before admission, he fell through a hatchway, striking his left knee. It became swollen and painful, but not enough so to keep him from walking about the house. In two weeks he resumed work, though the knee was not strong, and he worked only one day on account of pain experienced. From that time until his admission into the hospital it had grown steadily worse, and for a week before admission he was unable to use the limb at all. On examination the leg was found to be considerably flexed, but by careful manipulation it could be nearly straightened without pain. The knee was swollen, particularly at its outer and upper aspects, where it was also extremely tender. Exploratory puncture revealed blood and shreds of tissue, which were not examined microscopically. The patient suffered greatly, and the tenderness became more diffused, though neither swelling nor tender points could be discovered below the patella.

November 21st, the limb was suspended and a Martin's bandage applied. Extension with weight of four pounds was used.

November 26th, a cachectic appearance of the patient was observed. By December 3rd, swelling had extended five or six inches up the thigh.

December 4th, an exploratory incision, four inches long, over the outer condyle revealed large masses of morbid material and a large cavity in the femur filled with similar masses. There was no effusion into the joint, and no erosion of the cartilages, but the femur was roughened as far as the finger could reach. A circular amputation in upper third of thigh was made at once.

On December 5th there was considerable oozing; the dressing was changed and the lower drainage tube was removed.

On December 8th the last drainage tube and the sutures were removed.

December 21st, the patient was up and moved about on a wheel-chair. His highest temperature had been 99.5°F.

December 23rd, the patient evidently has been greatly relieved by the amputation, but the cachexia still continues and seems to be increasing.

Examination of the specimen discloses marked proliferation of cartilage and connective-tissue, especially in the vicinity of external condyle. In the cartilage are found numerous spicules of bone. Attached to the lower anterior external surface of the bone and extending into the neighboring tissues without distinct encapsulation is a moderately soft, fleshy mass about the size of a duck's egg. Numerous blood-spaces are enclosed in this mass. Beneath this new formation the bone is found to have been absorbed, and examination discloses the medullary cavity of the bone filled with a soft, pulpy sanguineous material in which the microscope reveals blood, fat, multiform and multinuclear cells. The knee-joint has not been invaded by the new formation.

Microscopical examination of sections removed from various portions of the tumor and its surroundings shows plastic inflammation of all the neighboring structures resulting in proliferation of cartilage, bone and connective-tissue. Characteristic sarcoma-structure is found throughout the new formation and also in fresh specimens removed from the medulla.

